# Decorin-armed oncolytic adenovirus promotes natural killers (NKs) activation and infiltration to enhance NK therapy in CRC model

**DOI:** 10.1186/s43556-024-00212-z

**Published:** 2024-11-01

**Authors:** Xue Li, Yuning Zhang, Zhuang Mao, Huiqiang Zhao, Hu Cao, Jingyi Wang, Wei Liu, Shiyun Dai, Yuefeng Yang, Yuanyuan Huang, Hua Wang

**Affiliations:** 1 College of Life Science, Anhui Medical University, Hefei, 230032 P.R. China; 2 Beijing Institute of Radiation Medicine, Beijing, 100850 China; 3 Department of Oncology, The Fifth Medical Center, Chinese PLA General Hospital, Beijing, 100071 China; 4 Department of Healthcare, The Second Medical Center & National Clinical Research Center for Geriatric Diseases, Chinese PLA General Hospital, Beijing, 100853 China; 5 Beijing Jingda Biotechnology Co. Ltd, Beijing, 102629 China; 6 Department of Experimental Medical Science, Ningbo No.2 Hospital, Ningbo, 315010 China

**Keywords:** Adoptive NK cell therapy, Oncolytic adenovirus, Decorin, Colorectal cancer

## Abstract

Colorectal cancer (CRC) is a prevalent malignant tumor of the gastrointestinal system, with the third and second highest incidence and mortality rates globally in 2020, respectively. Immunotherapy has developed rapidly in recent years. Natural killer (NK) cells have received increasing attention in the field of tumor immunotherapy due to their recognition and killing tumor cells without the limitations of major histocompatibility complexes. However, constraints within the tumor microenvironment that impede the infiltration and proliferation of NK cells result in poor efficacy of NK cell therapy for solid tumors. Oncolytic viral therapy is an immunogenic treatment with the potential to enhance anti-tumour immune responses and promote immune cell infiltration. In this study, we synergistically combine NK cells with an oncolytic adenovirus carrying Decorin (rAd.DCN) for the treatment of colorectal cancer (CRC) in a xenograft mouse model. By using Flow cytometry, real-time quantitative PCR and Calcein-AM release assay, we found that rAd.DCN could effectively promote proliferation, activation and degranulation of NK cells, up-regulate expression and secretion of NK cell killing activity-related factors, and enhance their killing activity. The efficacy is better than that of the blank control oncolytic virus rAd.Null. Combined treatment significantly inhibited tumor growth, increased the number of NK cells in peripheral blood, promoted the killing function of NK cells, and increased the expression levels of perforin and IFN-γ. At the same time, more NK cells were recruited to infiltrate tumor tissue. Our study established the feasibility of combination NK cells and oncolytic adenovirus application, thus expanding the scope of potentially curative treatments for NK cells in CRC.

**Supplementary Information:**

The online version contains supplementary material available at 10.1186/s43556-024-00212-z.

## Introduction

Colorectal cancer (CRC) stands as a prevalent malignancy worldwide, ranking as the third and second most common cancer incidence in men and women, respectively [1]. Many CRC patients receive an unsatisfactory prognosis as the disease was detected at a late stage, and cannot receive personalized treatment [2]. Despite significant reductions in overall morbidity and mortality over the past decades, the overall survival rate for patients with advanced colorectal cancer remains notably low [3]. Therefore, there is an urgent need to advance novel treatment strategies for colorectal cancer.

Tumor immunotherapy represents a therapeutic modality aimed at controlling and eliminating tumors by restoring the body’s normal anti-tumor immune response [4]. With continuous breakthroughs in tumor immunity technology, various strategies have emerged, including checkpoint inhibitor monoclonal antibodies [5], lymphocyte activating factors [6], tumor vaccines [7], oncolytic viruses [8], bispecific antibodies [9], and adoptive NK cell therapy [10]. NK cells, distinguished as the most lethal cytotoxic lymphocytes in the innate immune system, contribute to both innate and adaptive immunity, exerting potent antiviral and antitumor effects [11]. While NK cell infusion has shown initial success in treating hematological tumors, its efficacy against solid tumors is significantly hindered by challenges such as limited infiltration into deeper tumor regions and an immunosuppressive microenvironment [12]. Therefore, overcoming these limitations to enhance NK cell anti-tumor capacity is a critical issue.

Oncolytic viruses selectively replicate in tumor cells and lysis tumors, induce systemic and local anti-tumor immune responses by releasing tumor-associated antigens, enhance the tumor microenvironment, and thus promote infiltration of T-cells and other immune cells, paving the way for effective treatment of solid tumors with NK cell therapy [13, 14]. Decorin (DCN) exhibits the capability to inhibit tumor cell growth and metastasis, induce tumor cell apoptosis, and degrade tumor stroma. This occurs through specific down-regulation of epidermal growth factor (EGF) and vascular endothelial growth factor (VEGF) expressions, blocking TGF-β signaling, and attenuating Met and β-catenin signal pathways [15, 16]. An oncolytic adenovirus carrying DCN (rAd.DCN) has previously been developed, demonstrating inhibitory effects on breast cancer [17], as well as inhibiting tumor growth and lung metastasis of breast cancer [16] and bone metastasis of prostate cancer [18] in mouse models. And the inhibitory effects of rAd.DCN on tumor growth and metastasis were more significant than those of rAd.Null. Therefore, rAd.DCN was used in this manuscript to evaluate the antitumor effect of oncolytic virus combined with NK cells on CRC.

The combination approach of oncolytic viruses and cell therapy, two pivotal branches of immunotherapy, has received significant attention. However, the application of NK cells in combination with oncolytic virus therapy is still in the exploratory stage. Preclinical studies have demonstrated that the utilization of NK cells overexpressing CCR5 in combination with CCL5-armed oncolytic virus therapy exhibited superior efficacy compared to monotherapy in a mouse colon cancer model, and this combination approach significantly enhanced NK cell infiltration into the tumor microenvironment compared to wild-type virus [19]. Another preclinical study highlighted that the combination of an oncolytic virus expressing the IL15/IL15Rα complex with frozen, ready-to-use EGFR-CAR NK cells elicited potent antitumor responses in glioblastoma [20]. The addition of adoptive NKG2D-positive cells improved the effectiveness of oncolytic measles viral therapy in a murine model of hepatocellular carcinoma [21]. However, the antitumor effect of NK cells in combination with rAd.DCN on CRC has not been extensively explored.

In this study, we established a CRC model in immunodeficient NPG mice to evaluate the combined therapeutic effect of rAd.DCN and NK cells, as well as the enhanced anti-tumor effect of rAd.DCN on NK cells. We found that rAd.DCN could promote NK cell infiltration in tumor tissues and enhance NK cell proliferation and degranulation activity in peripheral blood. Our results demonstrate for the first time the inhibitory effect of NK cells combined with rAd. DCN on CRC growth, providing a reasonable combination strategy for CRC treatment.

## Results

### Peripheral blood derived natural killer cells (NK) were gradually activated by a feeder-free system in vitro

**Fig. 1:**
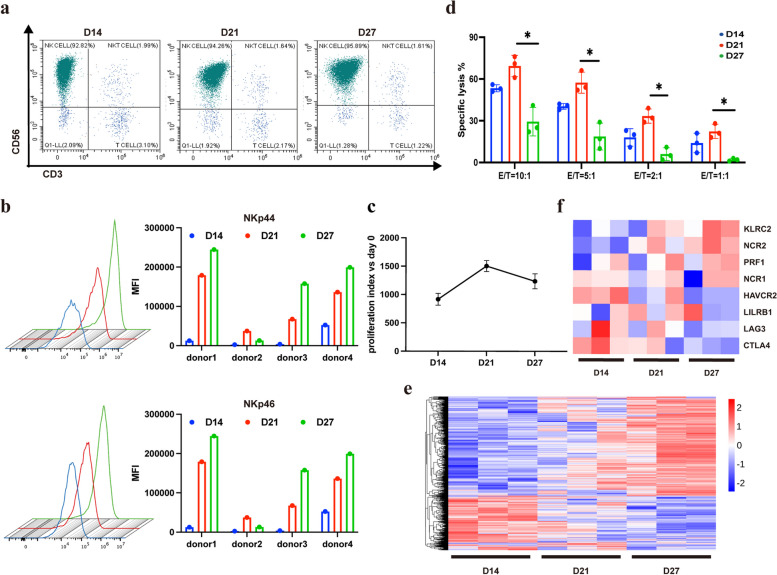
Peripheral blood derived natural killer cells (NK) were gradually activated by a feeder-free system in vitro. **a** The percentage of NK cells (CD3^−^CD56^+^) cultured in vitro at day 14, 21 and 27 was detected by using Flow cytometry. **b** Mean fluorescence intensity (MFI) of surface receptors NKp44 and NKp46 at different time points during NK cell expansion in vitro. **c** Growth curve of NK cells. **d** Comparison of killing activity of NK cells against HCT116 cells with different ratio of effector to target at different expansion time. **e** Heat map of differentially expressed genes. **f** Differentially expressed activating and inhibitory receptors of NK cells. One-way ANOVA was used for comparisons between multiple groups. All results are presented as the mean ± SD (*n* = 3)

In this study, peripheral blood mononuclear cells (PBMCs) were isolated from blood samples of healthy donors. Moreover, NK cells were activated and expanded by using a feeder-free culture system. We found that with the extension of the expansion time, the proportion of NK cells (CD3^−^CD56^+^), as shown in Fig. 1a, increased from 92.82% on day 14 to 94.26% on day 21, and finally reached 95.89% on day 27. Flow-cytometric analysis confirmed that expression of activated receptors NKp44 and NKp46 on the NK-cell surface increased with time of expansion (Fig. 1b). Simultaneously, the morphology of NK cells was observed and the proliferation index was calculated, and the results (Fig. 1c) showed that, on day 14, NK cells expanded rapidly, cell size gradually increased, and many cell colonies appeared. On day 21 of culture, the cell expansion rate tended to stabilize, and then with the prolongation of culture time, the cell expansion rate showed a decreasing trend. The proliferation index of day 21 was about 1500 when compared to day 0, while it decreased to 1100 on day 27. In addition, the killing activity of NK cells against HCT116 cells was also analyzed. The results showed that the killing activity of NK cells expanded for 21 days was significantly higher than that of NK cells cultured for 14 days and 27 days when the ratio of effector to target was the same (Fig. 1d). This may be due to exhaustion of NK cells caused by persistent activation in vitro. The main characteristics of NK cell exhaustion include impaired cytotoxicity, decreased secretion of cytokines, dysregulation of proliferation, metabolic dysfunction, and so on [22].

Next, RNA-seq analysis of NK cells at different time points was performed. The results revealed significant changes in gene expression at different time points of NK cell expansion in vitro. Compared to the day 14 group, there were 62 up-regulated and 86 down-regulated genes in the day 21 group, and 794 up-regulated and 386 down-regulated genes in the day 27 group. Additionally, there were 44 up-regulated genes and 27 down-regulated genes in the day 27group when compared with the day 21 group (Fig. 1e).

The cytotoxicity of NK cells depends on the integration and output of activation and inhibition signals mediated by NK cell surface receptors. The expression of activating and inhibitory receptors of NK cells at different culture times was analyzed. Activating receptors perforin (PRF1), NKG2C (KLRC2), NKp44 (NCR2), and NKp46 (NCR1) showed upregulation, while inhibitory receptors LILRB1, LAG3, CTLA4, and Tim3 (HAVCR2) exhibited downregulation with the extension of in vitro expansion (Fig. 1f).

GO function and KEGG pathway enrichment analyses highlighted associations with DNA replication origin binding, protein homodimerization activity, Ras guanyl-nucleotide exchange factor activity, nucleosome histone binding, and diacylglycerol binding (Supplementary Fig. 1a). DEGs were significantly enriched in Ras signaling pathways, complement and coagulation cascades pathways, hematopoietic cell lineage pathways, cell cycle pathways, and cytokine-cytokine receptor interaction pathways (Supplementary Fig. 1b).

In conclusion, we chose NK cells expanded for 21 days in vitro which possessed high killing activity and a high proliferation index, for subsequent animal experiments.

### rAd.DCN enhances cytotoxic activity of NK cells in vitro

**Fig. 2:**
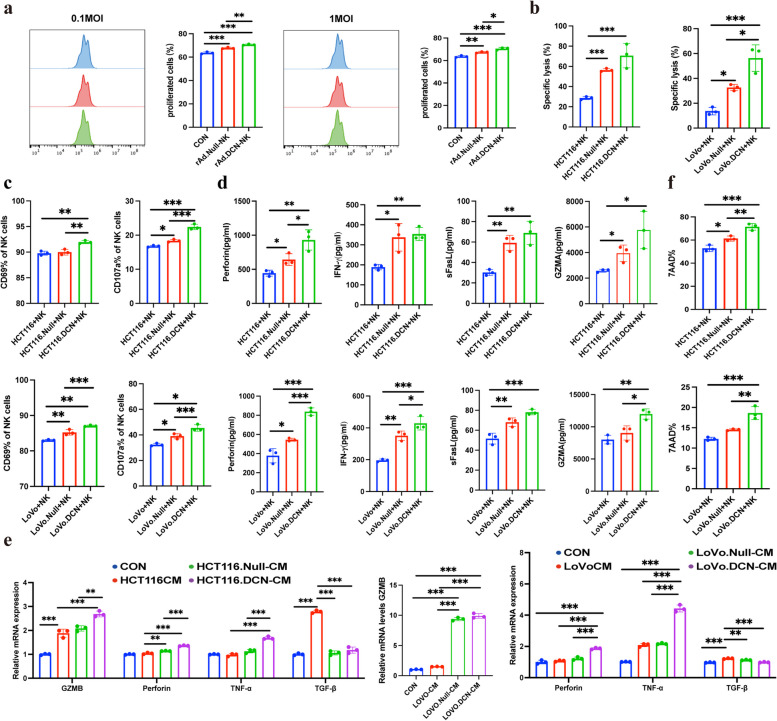
rAd.DCN enhances cytotoxic activity of NK cells in vitro. **a** NK cells were pre-stained with CFSE (5 µM) and then infected with Oncolytic Viruses (OVs) at MOIs of 0.1 and 1 for 72 h. Flow cytometry was utilized to analyze the proliferation of NK cells. **b** NK cells were co-incubated with rAd.Null or rAd.DCN (50 MOI) pretreated HCT116 cells or LoVo cells for 4 h, and a cytotoxicity assay was performed. The percentage of specific cell lysis of HCT116 and LoVo cells was analyzed. **c** NK cells were co-cultured with rAd.Null or rAd.DCN (50 MOI) pretreated HCT116 or LoVo cells for 4 h, and flow cytometry was used to detect the percentage of CD69 and CD107 positive NK cells. **d** Co-incubated supernatants were collected and assayed for perforin, IFN, sFasL, and GZMA secretion using the LEGENDplex™ Human CD8/NK Panel Kit. **e** NK cells were treated with the supernatant of control or OVs-infected HCT116 or LoVo cells for 48 h, and the expression of granzyme B, perforin, TNF-α, and TGF-β of NK cells was evaluated by qPCR assay. f HCT116 or LoVo cells pretreated with rAd.Null or rAd.DCN (50 MOI) were labelled using CFSE, and after co-culturing with NK cells for 4 h, the cells were collected and labelled with 7AAD, and apoptosis rate of target cells was detected by flow cytometry. One-way ANOVA was used for comparisons between multiple groups. All results are presented as the mean ± SD (*n* = 3). *P < 0.05; **P < 0.01, ***P < 0.001, ns means no significance

To investigate the ability of Oncolytic Viruses (OVs) to enhance NK cell function in vitro, the effect of OVs on NK cell proliferation was initially examined. NK cells, pre-stained with CFSE, were co-cultured with OVs at different MOIs. Results revealed that OVs at 0.1 and 1 MOI significantly affected NK cell proliferation, with rAd.DCN exhibiting a stronger effect than rAd.Null (Fig. 2a). To further elucidate the impact of rAd.DCN on the function of NK cells, HCT116 cells were infected with 50 MOI of rAd.DCN for 24 h and then co-incubated with NK cells for 4 h. The results indicated a significant increase in killing efficiency (Fig. 2b) and the percentage of NK cell surface activation markers CD69 and degranulation indicator CD107a in the virus-pretreated HCT116 cell group, with rAd.DCN outperforming rAd.Null (Fig. 2c). Additionally, analysis of the co-incubation supernatant revealed a significant increase in the secretion of perforin, IFN-γ, sFasL, and GZMA in HCT116 cells pretreated by rAd.DCN (Fig. 2d). The same results were also observed in LoVo cells (Fig. 2b-d).

To further validate the effect of virus-pretreated HCT116 and LoVo cells on NK cells, conditioned medium from HCT116 and LoVo cells infected with 50 MOI of rAd.DCN for 48 h were collected. Subsequent treatment of NK cells with this conditioned medium for 48 h resulted in a significant elevation in the expression of granzyme B, perforin, and TNF-α, along with a notable decrease in TGF-β expression, compared to the control group. The expression of granzyme B, perforin, and TNF-α in the rAd.DCN pretreated group was higher than in the rAd.Null pretreated group, with statistically significant differences between the two groups (Fig. 2e). In addition, the results of CFSE/7AAD cytotoxicity assay indicated that pretreatment with 50 MOI of rAd.DCN could significantly promote the pro-apoptotic effect of NK cells on HCT116 and LoVo cells (Fig. 2f). In summary, OVs pretreatment further stimulated NK cell proliferation, upregulated the expression of CD69 and CD107a, induced NK cells to secrete perforin, granzyme B, and TNF-α, inhibited TGF-β expression, and promoted apoptosis of HCT116 and LoVo cells, thereby promoting the anti-tumor effect of NK cells.

### Combined therapy with rAd.DCN and NK cells evoked stronger antitumor responses than monotherapy

**Fig. 3:**
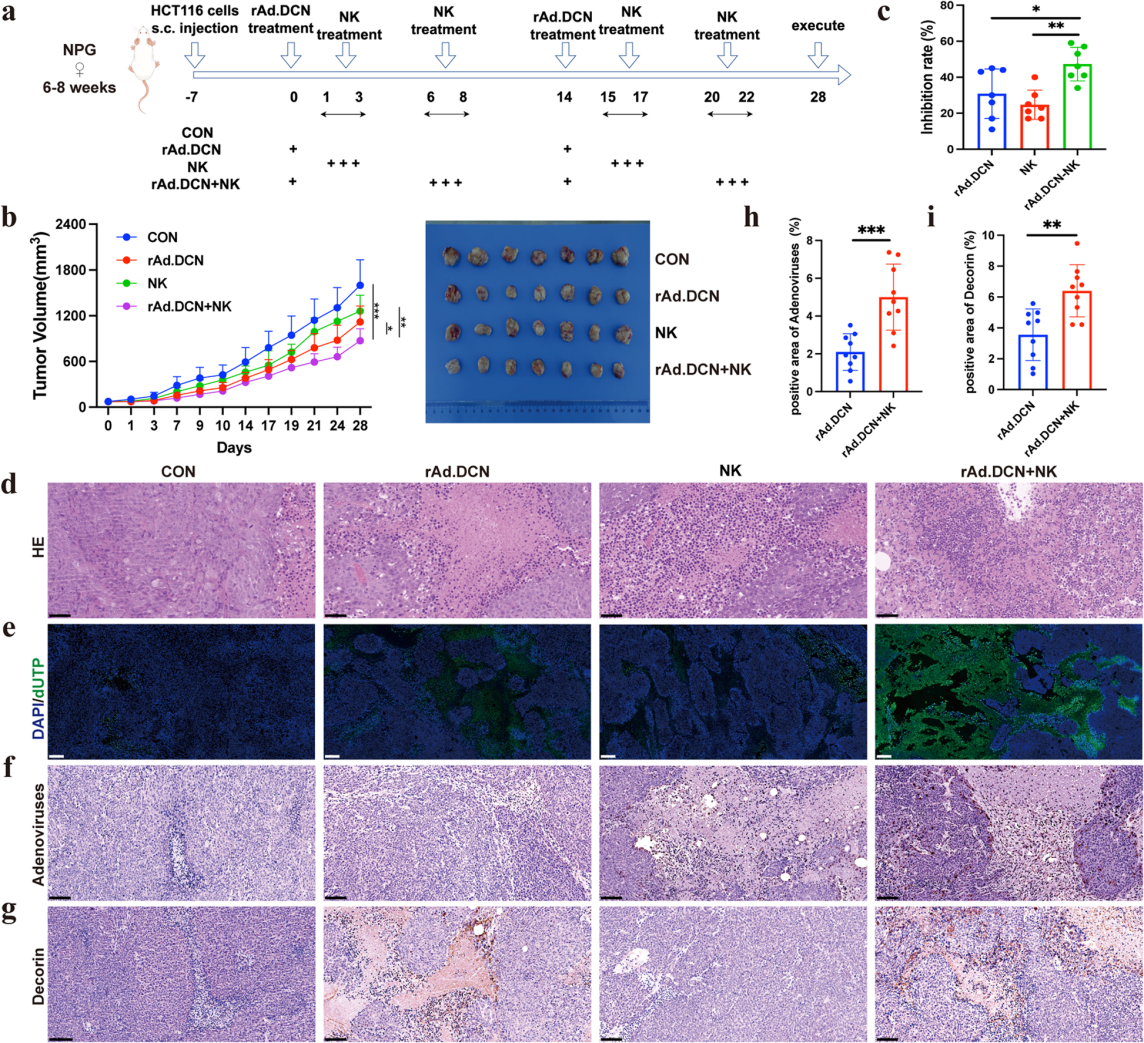
Combined therapy with rAd.DCN and NK cells evoked stronger antitumor responses than monotherapy (*n* = 11 per group). **a** Experimental timeline for in vivo studies (by Figdraw). **b** Tumor growth curves and representative tumor images (*n* = 7 for each group) at the experiment’s conclusion (day 28). **c** Tumor inhibition rate at the experimental endpoint (*n* = 7 for each group). **d** Representative images of HE staining. Scale bar = 50 µm. **e** Terminal deoxynucleotidyl transferase dUTP nick end labeling (TUNEL) for assessing tumor tissue apoptosis. Scale bar = 200 µm. **f** Representative images of adenoviral distribution and **g** Decorin expression in tumor tissues. **h** Statistical results of positive area ratio for adenovirus and **i** Decorin. Scale bar = 50 µm. Comparisons between the two groups were made using the unpaired t-test. All results are presented as the mean ± SD. * *p* < 0.05; **p < 0.01; ***p < 0.001

The combination of immunotherapy and virus therapy has emerged as a novel and promising strategy for cancer treatment. To evaluate the efficacy of the combination therapy involving NK cells and rAd.DCN, a subcutaneous xenograft model of human colon cancer was established. Treatment commenced when the tumor volume reached approximately 70 mm^3^, as depicted in Fig. 3a. Monotherapy with rAd.DCN and NK cells demonstrated moderate antitumor activity, with tumor growth inhibition rates of 30.8% and 24.7%, respectively, compared to the control group. The combination therapy, however, resulted in a more remarkable response, reducing tumor volume by approximately 47.3% compared to the control group (Fig. 3b & c).

HE staining revealed necrotic lesion areas in tumor tissue across all four groups. Nevertheless, significantly more necrotic areas and inflammatory infiltrations were observed in both monotherapy and combined therapy groups (Fig. 3d). TUNEL staining indicated noticeable apoptosis/death in both monotherapy and combined therapy groups, with minimal occurrence in the control group. Importantly, the rAd.DCN and NK combined group induced apoptosis/death more effectively than the monotherapy group (Fig. 3e). These findings suggest that the combination of rAd.DCN and NK enhances the in vivo anti-tumor effect compared to monotherapy.

Targeting tumor cells and producing therapeutic proteins are pivotal aspects of oncolytic viruses (OVs)-mediated gene therapy. In this study, we demonstrated that intratumorally injected adenovirus remained detectable until day 28 (Fig. 3f & h) and efficiently produced Decorin in tumor tissue (Fig. 3g & i).

### Local administration of rAd.DCN augments NK cell cytotoxic activity and promotes NK cell infiltration in tumor microenvironment

**Fig. 4:**
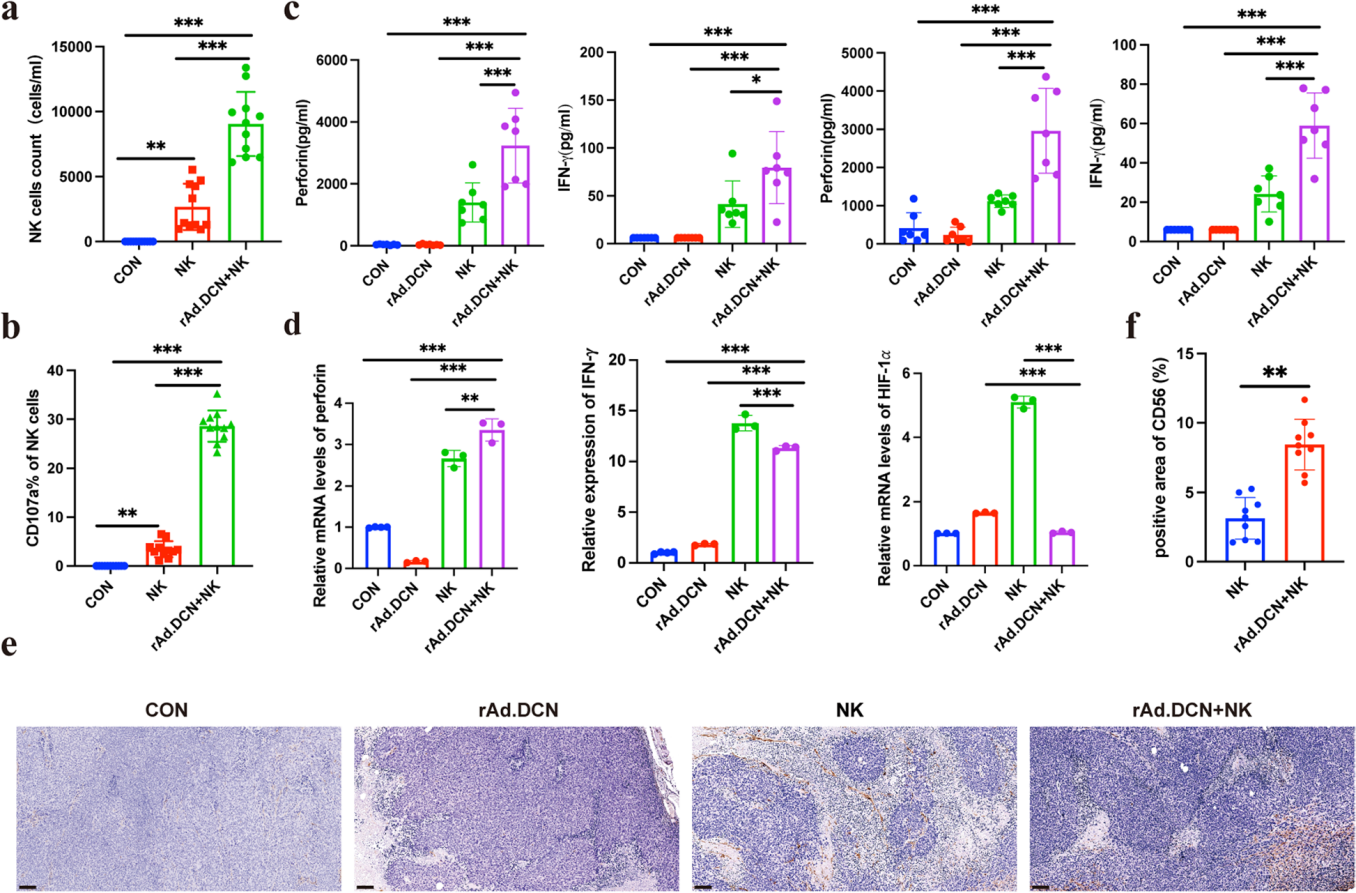
Local administration of rAd.DCN augments NK cell cytotoxic activity and promotes NK cell infiltration in tumor microenvironment. **a** NK cell counts and **b** CD107a expression in peripheral blood detected by flow cytometry at 24 h after the first NK infusion (*n* = 11). **c** Concentration of Perforin and IFN-γ in serum analyzed by LEGENDplex™ Human CD8/NK Panel Kit at 24 h after NK cell infusion in the first and second treatment cycle (*n* = 7). **d** Quantitative PCR assay for the expression of perforin, IFN-γ, and HIF-1α in tumor tissues (*n* = 3). One-way ANOVA was used for comparisons between multiple groups. **e** Immunohistochemical staining for the distribution of CD56-positive cells. **f** Statistical results of positive area ratio for CD56 (*n* = 9). Comparisons between the two groups were made using an unpaired t-test. Scale bar = 100 µm. Data presented as mean ± SD. *p < 0.05; **p < 0.01; ***p < 0.001

To elucidate the mechanism behind the heightened anti-tumor activity induced by rAd.DCN, we assessed the number and activation status of NK cells in peripheral blood using flow cytometry 24 h post-NK cell infusion. Results revealed a significant increase in NK cell count in the rAd.DCN + NK combination group compared to the NK group (Fig. 4a), accompanied by a notable elevation in CD107a expression within NK cells in the combined therapy group (Fig. 4b). Furthermore, the concentration of perforin and IFN-γ in the serum, measured 24 h after NK cell infusion in the first and second treatment cycles, was higher in the rAd.DCN + NK combined group than in the two monotherapy groups (Fig. 4c).

Quantitative PCR analysis of tumor tissues demonstrated that rAd.DCN + NK combination treatment significantly upregulated perforin and IFN-γ expression and downregulated HIF-1α expression compared to rAd.DCN or NK monotherapy groups (Fig. 4d). Immunohistochemical analysis of CD56 expression in tumor tissues revealed more CD56-positive staining in the NK and rAd.DCN + NK groups, with no CD56-positive staining in the CON or rAd.DCN groups. Importantly, the average optical density of CD56-positive staining was higher in the rAd.DCN + NK combination treatment group than in the NK group (Fig. 4e-f). These findings suggest that rAd.DCN promotes NK cell infiltration into tumor tissues and enhances NK cell cytotoxic activity.

### The balance between anti-virus immunity and anti-tumor immunity of NK cells in combination therapy

**Fig. 5:**
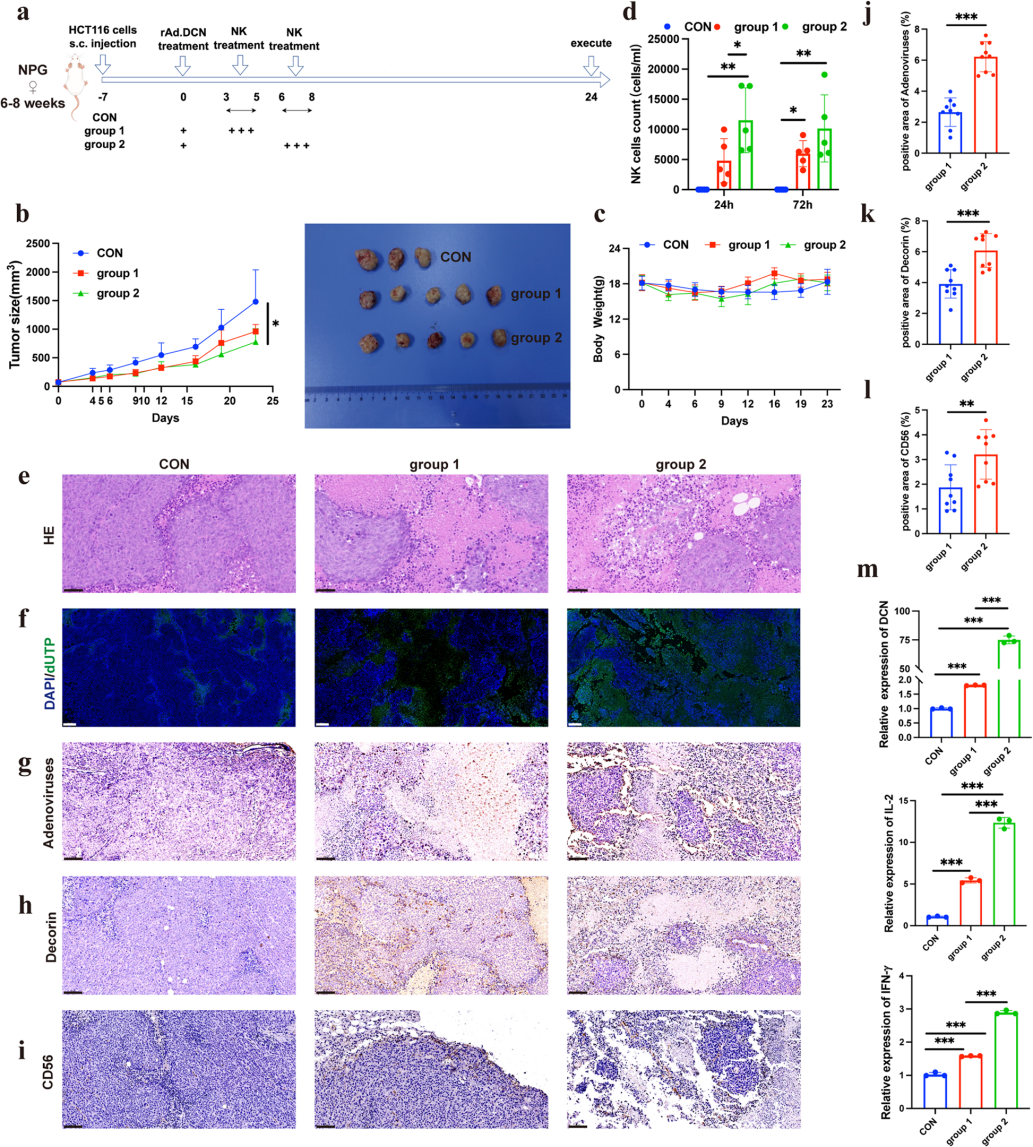
The balance between anti-virus immunity and anti-tumor immunity of NK cells in combination therapy (*n* = 5 per group). **a** Treatment protocol for HCT116 tumor-bearing NPG mice (by Figdraw). In group 1, NK infusion was started on day 3 after rAd.DCN injection, and in group 2, NK infusion was started on day 6 after rAd.DCN injection. **b** Tumor growth curves and images of tumor tissues from sacrificed mice at day 24 (*n* = 3 for the control group or *n* = 5 for rAd.DCN + NK group). Tumor volumes were subjected to analysis using a two-way repeated-measure ANOVA. **c** Body weights of mice during the 24-day course of treatment (*n* = 3 for the control group or *n* = 5 for rAd.DCN + NK group). **d** Analysis of NK cell counts in the peripheral blood of mice using flow cytometry (*n* = 3 for the control group or *n* = 5 for rAd.DCN + NK group). One-way ANOVA was used for comparisons between multiple groups. **e** HE staining of tumor tissue at the experimental endpoint. Scale bar = 50 µm. **f** TUNEL staining of tumor tissues. Scale bar = 200 µm. Immunohistochemistry staining for **g** adenovirus, **h** Decorin, and **i** CD56 distribution in the tumor tissues. Scale bar = 50 µm. **j** Statistical results of positive area ratio for adenovirus, **k** Decorin and **l** CD56 (*n* = 9). Comparisons between the two groups were made using an unpaired t-test. **m** qPCR detection of Decorin, IL-2, and IFN-γ expression in tumor tissues (*n* = 3). One-way ANOVA was used for comparisons between multiple groups. Data are presented as mean ± SD. *p < 0.05; ***p < 0.001

NK cells play a role in virus-induced inflammation by targeting and destroying virus-infected host cells and producing IFN-γ. Therefore, they may have a negative impact on OV therapy by inhibiting the spread of OVs in tumors and limiting the degree of virus-mediated oncolysis [23]. Therefore, it is necessary to optimize the time interval between OV and NK cell administration to balance the unwanted early NK cell clearance of OV or OV-infected tumor cells with the required late anti-tumor response of NK cells. To optimize treatment intervals between rAd.DCN and NK cells, HCT116 tumor-bearing NPG mice were divided into three groups: CON group, rAd.DCN + NK group 1 (NK infusion from the 3rd day after rAd.DCN injection), and rAd.DCN + NK group 2 (NK infusion from the 6th day after rAd.DCN injection). The treatment scheme is shown in Fig. 5a. Results showed that both rAd.DCN + NK group 1 and group 2 significantly inhibited tumor growth compared to the control group. Notably, rAd.DCN + NK group 2 exhibited smaller tumor sizes than group 1 from day 16 after HCT116 inoculation, suggesting better inhibition of tumor growth (Fig. 5b). There were no significant differences in body weight between the three groups during treatment (Fig. 5c).

Peripheral blood analysis at 24 h and 72 h after NK administration revealed a higher number of NK cells in the peripheral blood of rAd.DCN + NK group 2 compared to group 1 (Fig. 5d).

Histopathological analysis, including HE staining, TUNEL staining, and detection of adenovirus, Decorin expression, and NK distribution, showed that rAd.DCN + NK group 2 induced more necrotic lesions, stronger apoptosis, and increased presence of adenovirus, Decorin, and NK cells compared to group 1 (Fig. 5e-l).

Furthermore, in order to determine the expression levels of Decorin, IL-2, and IFN-γ in tumor tissue, a qPCR assay was conducted. Eventually, a notable increase in the expression of Decorin, IL-2, and IFN-γ was observed in group 2 of the rAd.DCN + NK cohort in comparison to group 1 (Fig. 5m).

### Combined therapy of rAd.DCN and NK cells does not increase liver toxicity in xenograft model

**Fig. 6:**
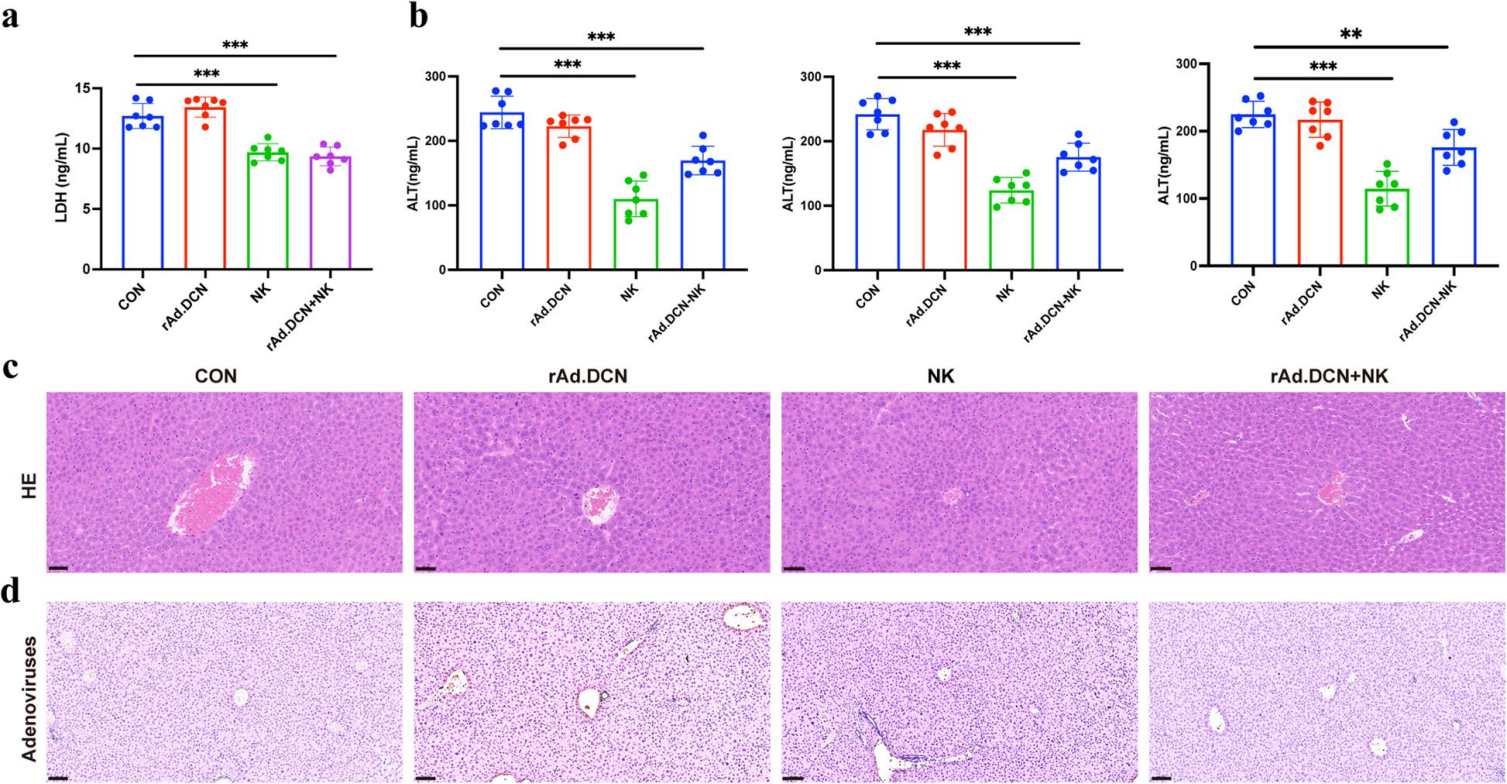
Combined therapy with rAd.DCN and NK cells does not increase the toxicity in xenograft model. **a** Results of serum LDH assay in mice at the endpoint of the experiment. **b** Detection of ALT in serum 24 h after NK cell infusion in the first and second treatment cycles and at the end of the experiment. One-way ANOVA was used for comparisons between multiple groups. **c** Representative images of HE staining of liver tissues. Scale bar = 50 µm. **d** Representative images of histochemistry staining of adenovirus in liver tissues. Scale bar = 100 µm. Data are presented as mean ± SD (*n* = 7). **p < 0.01; ***p < 0.001

Immunotherapy has the potential for serious side effects, necessitating a thorough assessment of the safety profile associated with rAd.DCN and NK combination therapy. Comparative analysis with the control group revealed no significant differences in weight and blood parameters (WBC, RBC, Hb, platelet, neutrophil, and monocyte) among mice in monotherapy and combined treatment groups (Supplementary Fig. 2a & 2b). Concurrently, cell injury index LDH activity and liver function index ALT in tumor-bearing mice have been evaluated. In comparison to the control group, serum LDH and ALT activity in the rAd.DCN group showed no change, while they were significantly reduced in both the NK group and the combined group. These findings suggest that neither monotherapy nor combination therapy adversely affects liver function (Fig. 6a & b). Additionally, no evident histopathological changes were observed in the liver tissues of the monotherapy and combined therapy groups (Fig. 6c). Further examination of adenovirus distribution revealed no presence of adenovirus in liver tissue (Fig. 6d). These results highlight the controlled safety profile of combined therapy.

## Discussion

Natural Killer (NK) cells are pivotal components of innate immunity, forming the frontline defense against pathogens and malignant tumors. Activated NK cells exhibit rapid cytotoxicity against infected or cancerous cells without prior sensitization and are not confined by the MHC [24, 25]. Compared to T cells, NK cells have demonstrated superior anti-cancer stem cells (CSCs) capabilities in recognizing low levels of MHC-I expression. However, CSC escape also occurs during NK cell therapy. Given the immune evasion and specific characteristics of CSCs, novel ideas based on NK adoptive cell therapy have been proposed to enhance NK cells’ antitumor capabilities to effectively eradicate all tumor cells. This includes chimeric antigen receptor-NK (CAR-NK) therapy, and NK cell therapy combined with other therapies, such as targeted therapy, T cell therapy, chemotherapy, or radiotherapy [26]. Antibody-dependent cell-mediated cytotoxicity (ADCC) by NK cells is a key mechanism for several clinically successful tumor-targeting therapeutic antibodies [27]. Some immunosuppressive factors secreted by cancer cells, such as indoleamine 2,3-dioxygenase 1 (IDO1) and tryptophan 2,3-dioxygenase 2 (TDO2), could suppress functional NK cells mediated ADCC. Thus, the inhibitor targeting these factors showed a promising effect on reversing podoplanin-positive cancer-associated fibroblasts-induced suppression of NK cells mediated ADCC [28]. Furthermore, NK cells act as regulatory cells by secreting various cytokines that initiate and enhance adaptive immune responses against tumors or infected cells [24, 25]. The clinical application of cancer therapy has demonstrated the superiority of NK cells, highlighting their potential in cancer treatment [29]. The cytotoxic activity of NK cells relies on the integration and output of activating and inhibitory signals mediated by receptors on the NK cell surface. In this study, we analyzed the receptor expression pattern of expanded NK cells in vitro through flow cytometry and RNA-seq. The results indicated an upregulation of activating receptors, including perforin, NKG2C, NKp44, and NKp46, and a downregulation of inhibitory receptors such as LILR, LAG3, CTLA4, and Tim3 with the extension of the expansion time. But the results of the proliferation index of NK cells in vitro suggested that the expansion multiple of NK cells was stable at day 21 and decreased at day 27. Meanwhile, we also detected the killing activity of NK cells in this study, and we found that the killing activity of NK cells at day 21 was significantly higher than that at day 14 and day 27. These results inferred that too long expansion time of NK cells in vitro would cause the exhaustion of NK cells and lead to the decline of their functions, but the potential molecular regulation mechanism needed further exploration. Consequently, NK cells amplified for 21 days in vitro were chosen for subsequent experiments, displaying higher NK cell activity.

Despite the promise of NK cells in cancer therapy, their efficacy against solid tumors is limited by factors such as restricted proliferation and persistence in vivo, along with the immunosuppressive tumor microenvironment. In addition, NK cell-associated cytotoxicity may also be impaired by stromal cells. For example, cancer-associated fibroblasts, monocytes, macrophages and other immune cells [30]. Strategies to overcome these limitations, including reversing immunosuppression, removing functional inhibition of NK cell, and promoting infiltration into tumor cells, could enhance the efficacy of NK cell immunotherapy [31]. In this study, we evaluated the synergistic antitumor effects of combining NK cells with the oncolytic adenovirus rAd.DCN. The combined therapy effectively inhibited tumor growth, induced tumor cell apoptosis, and increased NK cell numbers in peripheral blood and tumor tissue. The expression of CD107a, a marker representative of activated cytotoxic lymphocytes, was higher in the combined group. Additionally, the expression levels of perforin and IFN-γ, associated with NK cell cytotoxic activity, were significantly upregulated, while the expression level of HIF-1α, linked to the immunosuppressive microenvironment, was significantly downregulated in tumor tissue in the combined therapy group. In vitro assays further demonstrated that rAd.DCN could promote NK cell proliferation and enhance its killing function. The combination of NK and rAd.DCN had a stronger cytotoxic effect and a more potent killing effect on HCT116 and LoVo cells. Overall, rAd.DCN promoted the proliferation and persistence of NK cells and partially reversed the immunosuppressive tumor microenvironment, enhancing NK cell tumor-killing efficacy.

Notably, the effect of rAd.DCN on enhancing the cytotoxic function of NK cells was more effective than that of rAd.Null. This enhanced effect may be attributed to the role of Decorin carried by rAd.DCN. Decorin, in addition to being a component of the extracellular matrix, has crucial biological functions affecting cell proliferation, differentiation, apoptosis, angiogenesis, and tumor formation [32–34]. The current study extends previous research on the inhibitory effect of rAd.DCN on bone metastasis of breast cancer [17].

In addition to the enhancement of NK cells in proliferation, persistence and cytotoxic function combined with rAd.DCN, as well as the reversal of immunosuppressive TME, the mechanism by which rAd.DCN promotes the efficacy of NK cells may also lie in its direct lyse of tumor cells and the inhibitory effect of decorin genes on tumors by targeting and blocking pivotal signaling pathways, such as TGF-β, Met, wnt/β-catenin, and VEGF, which are associated with tumor immune escape and metastasis [35]. Moreover, expression of chemokines and adhesion molecules within the tumor microenvironment induced by OVs may additionally improve the antitumor effect via promoting the immune cell infiltration [36], which has been confirmed in our experimental results that rAd.DCN enhances NK cell infiltration into tumor tissue. However, there are some deficiencies in the verification at the molecular level, which need to be verified by later studies.

As many research has shown that oncolytic virus therapy can enhance the therapeutic effects of adoptive cell therapy [20, 37, 38], which is consistent with our findings in this study on the CRC xenograft model, the combination of rAd.DCN and NK cell therapy may have promising clinical application prospects, intratumoral administration of rAd.DCN via gastrointestinal endoscopy in combination with adoptive infusion of NK cells will become possible. However, potential challenges exist that hinder optimal performance in combination therapies. This could include addressing how to enhance rAd.DCN delivery efficiency in vivo and how to avoid NK cell immune rejection, as well as how to reach a proper balance between rapid virus elimination or clearance of OV virus-infected cells by NK cells and subsequent anti-tumor NK cell response, etc.

NK cells have the ability to quickly remove invading pathogens, such as viruses or transformed cells, such as cancer cells [39]. Under the background of oncolytic virus therapy, NK cells are known to mediate antiviral and antitumor responses [40]. However, the proper balance between the rapid virus elimination or clearance of OV virus-infected cells by NK cells and the later anti-tumor NK cell response is still not fully understood [41]. It should be noted that the efficacy of oncolytic virus therapy depends largely on this balance [42]. Strategies are needed to address these challenges, such as reversing the immunosuppressive microenvironment and carefully timing sequential administration to achieve optimal synergy [43]. In this research, we investigated the optimal interval between intratumoral injection of rAd.DCN and intravenous injection of NK cells, finding that a slightly longer interval produced more favorable outcomes. In addition, safety evaluations showed no observed cardiotoxicity or hepatotoxicity in the combination therapy group, indicating the safety and efficacy of NK cells combined with rAd.DCN in the treatment of colon cancer.

In conclusion, our study is the first to find that the combined use of NK cells and rAd.DCN exert synergistic anti-tumor effects, with rAd.DCN enhancing the number of NK cells in mouse peripheral blood and promoting the infiltration, survival, and cytotoxic function of NK cells in tumor lesions. These findings highlight the potential of rAd.DCN as an ‘adjuvant’ for NK cell therapy, presenting a novel strategy in cancer immunotherapy and laying the foundation for further clinical research on combined therapy for colon cancer. However, limitations exist in our research, including the use of NPG mice with human tumors and human NK cells. The defective normal immune system in these mice hinders the evaluation of other immune cells associated with therapeutic effects, limiting the evaluation of real-world immune response activity. Validation of these findings in immunocompetent mice, further exploration of the underlying mechanisms, and investigation of potential combinations with checkpoint blocking antibodies will be our future research directions.

## Materials and methods

### Cell lines and cell culture

Human colorectal cancer cell lines HCT116 and LoVo were procured from the American Type Culture Collection (ATCC, Manassas, VA, USA). HCT116 cells were cultured in McCoy’s 5A complete medium (Gibco, Thermo Fisher Scientific, Waltham, MA, USA) supplemented with 10% fetal bovine serum (FBS; Gibco). LoVo cells were cultured in Ham’s F-12 K complete medium (Gibco, Thermo Fisher Scientific, Waltham, MA, USA) supplemented with 10% fetal bovine serum (FBS; Gibco).

Isolation and Culture of Primary Human NK Cells: Primary human NK cells were isolated from peripheral blood mononuclear cells (PBMCs) and assessed for purity by flow cytometry using PerCP/Cyanine5.5 anti-human CD3 (344808, Biolegend, CA, USA) and APC anti-human CD56 (362504, Biolegend, CA, USA). NK cells were maintained in NK MACS Medium (Miltenyi Biotech, Bergisch Gladbach, Germany) with 2000 IU/mL IL-2 (Sihuan, Beijing, China), 100 ng/mL IL-15 (Novoprotein, Suzhou, China), and 5% HPL (Biological Industries, Germany). Peripheral blood was collected from healthy adult donors, and all experimental procedures were approved by Beijing Yuho Hospital (IRB-AF-27-00). Written informed consent was obtained from all donors participating in the study.

### Adenoviruses

Oncolytic adenoviruses were constructed using a simplified system for generating oncolytic adenovirus vectors carrying a single transgene, as previously described [44]. rAd.DCN, an oncolytic adenovirus expressing decorin under the control of the cytomegalovirus (CMV) promoter, was employed. The replication of adenoviruses was regulated by TERTp, situated upstream of E1A. The study utilized rAd.Null, an oncolytic adenovirus lacking any exogenous gene, as a control vector.

### Exploration of biological differences in NK cells during in vitro expansion

NK cells from three different donors, cultured in vitro, were collected on days 14, 21, and 27. Purified NK cells (CD3^−^CD56^+^ cells) were obtained by negative sorting with CD3 magnetic beads (Miltenyi Biotech, Bergisch Gladbach, Germany) through MACS. RNA-seq analysis was conducted on purified NK cells at each time point, and differentially expressed genes (DEGs) were identified based on |log2 (Fold Change) |> 2 and *P* < 0.05. Subsequent GO function and KEGG pathway enrichment analyses were performed on the DEGs.

### Cell counting

Cells were collected at day 0, 14, 21 and 27 of expansion, stained with AO/PI dye (Inno-Alliance Biotech, USA) for 2 min and the cell counts were performed using an automated cell counter (Countstar, Inno-Alliance Biotech, USA).

### CFSE cell labeling

To assess the impact of rAd.DCN on NK cell proliferation, NK cells were labeled with CFSE (423801, Biolegend, CA, USA) at a final concentration of 5 μM and infected with 1 or 0.1 MOI rAd.DCN. After 72 h of infection, NK cells were collected and proliferation was assessed by flow cytometry.

### Preparation of conditioned medium

HCT116 and LoVo cells were infected with 50 MOI of rAd.Null or rAd.DCN, or an equal amount of PBS. After 4 h incubation, the virus-containing medium was replaced, cells were washed thrice with PBS, and 10 mL of fresh medium was added. Cells and supernatant were collected 48 h post-infection, subjected to three cycles of freeze–thaw between 37 °C and -80 °C, and centrifuged at 10,000 rpm for 5 min. The virus-containing supernatant was frozen and stored at -80 °C for subsequent use.

### Cytotoxicity assay

A standard 4-h Calcein-AM release assay was conducted as previously described [45]. The percentage of specific cell lysis was calculated using the formula: 100 × (cpm experimental release – cpm spontaneous release) / (cpm maximal release – cpm spontaneous release).

### RNA isolation, reverse transcription, and real-time quantitative PCR (qPCR)

Total RNA was isolated from NK cells or tumor samples, followed by cDNA synthesis using the Evo M-MLV RT Mix Kit (Acclurate, China). qPCR was performed using a qPCR SYBR Green I Master kit (Yeasen, China) and a CFX Connect Real-Time System (Bio-Rad, USA). Primer sequences for real-time PCR are listed in Table 1. The Data were normalized to β-actin and expressed as relative mRNA levels.

**Table 1:** Primer sequences for qPCR

Gene (Human)	Sequences (5′ to 3′)
β-actin	Forward: ACAGAGCCTCGCCTTTGC
	Reverse: GATATCATCATCCATGGTGAGCTGG
IL-2	Forward: AGAACTCAAACCTCTGGAGGAAG
	Reverse: GCTGTCTCATCAGCATATTCACAC
DCN	Forward: GCTCTCCTACATCCGCATTGCT
	Reverse: GTCCTTTCAGGCTAGCTGCATC
IFN-γ	Forward: GAGTGTGGAGACCATCAAGGA
	Reverse: GGACATTCAAGTCAGTTACCGAA
Perforin	Forward: ACTCACAGGCAGCCAACTTTGC
	Reverse: CTCTTGAAGTCAGGGTGCAGCG
GZMB	Forward: CGACAGTACCATTGAGTTGTGCG Reverse: TTCGTCCATAGGAGACAATGCCC
HIF-1α	Forward: TATGAGCCAGAAGAACTTTTAGGC Reverse:CACCTCTTTTGGCAAGCATCCTG

### CFSE/7AAD cytotoxicity assay

NK cell-mediated cytotoxicity to oncolytic virus pretreated CRC cells was assessed by flow cytometry using the CFSE/7AAD cytotoxicity assay. HCT116 and LoVo cells pretreated or not with rAd.Null, rAd.DCN were labeled with 5 µM CFSE (423,801, Biolegend, CA, USA) for 20 min at 37 ℃. The cells were then mixed with target cells at an effector-to-target ratio of 5:1 (E: T = 5:1) and incubated for 4 h at 37℃. The cell mixture was then incubated with 7-AAD (00699350, Invitrogen CA, USA) for 15 min and detected by Flow cytometry (CytoFLEX).

### Establishment and treatment of transplanted tumor model of colon cancer

#### Animal experimental protocol

The animal experimental protocol was approved by the Institutional Animal Care and Use Committee of the Laboratory Animal Center (IACUC-DWZX-2023-514). 5-week-old female NPG mice weighing 18-24 g were obtained from Beijing Vitalstar Biotechnology Co., Ltd. (Beijing, China). After a one-week acclimation period, HCT116 cells (5 × 10^6^ cells/mouse) were injected into the right forelimb axilla of NPG mice.

#### Evaluation of therapy effect

Tumor-bearing mice were randomly divided into four groups (*n* = 11/group) at day 6 after HCT116 inoculation (named day 0): control group, rAd.DCN group, NK group, and rAd.DCN + NK group. Control group, mice were treated with PBS via intratumor injection at day 0 and day 14, and PBS via tail vein at day 6, 7, 8, 20, 21, and 22; rAd.DCN group, mice were treated with rAd.DCN (2 × 10^8^ PFU/mouse) via intratumor injection at day 0 and day 14; NK group, mice were treated with NK (1 × 10^7^ cells/mouse) via tail vein at day 1, 2, 3, 15,16 and 17; rAd.DCN + NK group, mice were treated with rAd.DCN (2 × 10^8^ PFU/mouse) via intratumor injection at day 0 and day 14, and treated with NK (1 × 10^7^ cells/mouse) via tail vein at day 6, 7, 8, 20, 21 and 22. The treatment regimen for each group was established, and tumor volumes were measured using digital calipers. Tumor size calculations were based on the formula: tumor volume = width^2^ × length/2.

#### Optimization of therapy intervals

HCT116-loaded NPG mice were randomly divided into three groups: CON group, mice were treated with PBS via intratumor injection at day 0, and PBS via tail vein at day 3, 4 and 5; rAd.DCN + NK group 1, mice were treated with rAd.DCN (2 × 10^8^ PFU/mouse) via intratumor injection at day 0, and treated with NK (1 × 10^7^ cells/mouse) via tail vein at day 3, 4 and 5; and rAd.DCN + NK group 2. mice were treated with rAd.DCN (2 × 10^8^ PFU/mouse) via intratumor injection at day 0, and treated with NK (1 × 10^7^ cells/mouse) via tail vein at day 6, 7 and 8. These groups received specific treatments to optimize therapy intervals.

### Flow cytometry

#### Phenotypic changes during in vitro expansion

NK cells collected on days 14, 21, and 27 were labeled with APC anti-human CD56 (362504, Biolegend, CA, USA), PerCP/Cyanine5.5 anti-human CD3 (344808, Biolegend, CA, USA), PE-Anti-NKp44 (325108, Biolegend, CA, USA), PE-Anti-NKp46 (331908, Biolegend, CA, USA), and PE-Anti-IgG (1400114, Biolegend, CA, USA). Flow cytometry analysis (CytoFLEX) was conducted to assess phenotypic changes in NK cells.

#### Activation and degranulation indicators

NK cells were collected and incubated with PE anti-human CD69 (310905, Biolegend, CA, USA) and PE/Cyanine7 anti-human CD107a (328617, Biolegend, CA, USA). Flow cytometry analysis was performed to detect activation and degranulation indicators.

#### Peripheral blood analysis

Peripheral blood collected 24 h after NK treatment was stained with antibodies, including FITC anti-mouse CD45 (328617, Biolegend, CA, USA), PE anti-human CD45 (304008, Biolegend, CA, USA), APC anti-human CD56 (362504, Biolegend, CA, USA), PerCP/Cyanine5.5 anti-human CD3 (344808, Biolegend, CA, USA), and PE/Cyanine7 anti-human CD107a (328617, Biolegend, CA, USA). Flow cytometry analysis was conducted using FlowJo version 10 software.

### Assessment of liver toxicity

#### Serum analysis

At the endpoint, serum was harvested to measure lactate dehydrogenase (LDH) and alanine transaminase (ALT) levels, serving as indicators of potential toxicity in vivo.

### Cytokine measurement

#### Perforin and IFN-γ levels

Perforin and IFN-γ levels in the serum were measured using LEGENDplex™ Human CD8/NK Panel (741186, Biolegend, California, USA) and analyzed via flow cytometry.

#### Co-incubated supernatants

Supernatants of perforin, IFN-γ, sFasL, and GZMA were determined by LEGENDplex™ Human CD8/NK Panel (741186, Biolegend, California, USA) and analyzed via flow cytometry.

### Histopathological analysis and immunohistochemistry

#### Histopathological examination

Tumor and liver tissues harvested at the endpoint were processed and stained with hematoxylin and eosin (H&E) for histopathological analysis.

#### Apoptosis detection

Apoptosis in tumor lesions was examined by terminal deoxynucleotidyl transferase UTP nick end labeling (TUNEL) according to the manufacturer’s instructions (Promega, Madison, WI).

#### Immunohistochemistry

Tumor and liver tissues were stained with rabbit anti-human adenovirus antibody (ab6982, Abcam, Cambridge, UK) and rabbit anti-human Decorin antibody (A1669, ABclonal, Wuhan, China) to detect the distribution of adenoviruses and the expression of Decorin. Tumors were stained with rabbit anti-human CD56 antibody (ab313779, Abcam, Cambridge, UK) to detect CD56 infiltration. The results of immunohistochemistry were quantitatively analyzed by ImageJ and the percentage of positive staining area (area %) of adenovirus, Decorin and CD56 were calculated.

### Statistical analysis

Statistical analysis was performed using GraphPad Prism software (version 9.0). All data are presented as means ± standard deviation (SD). Tumor volumes were analyzed using a two-way repeated-measure ANOVA. For comparisons between multiple groups, one-way ANOVA was employed, and unpaired t-tests were utilized for comparisons between two groups. A significance level of *p* < 0.05 was considered statistically significant.

## Supplementary Information

Supplementary Material 1.

Supplementary Material 2.

## Data Availability

All data generated or analysed during this study are included in this published article and its supplementary information files. The data supporting the findings of this study are available from the corresponding author upon reasonable request.
