# Supplementary figures and images for: Decorin-armed oncolytic adenovirus promotes natural killers (NKs) activation and infiltration to enhance NK therapy in CRC model
Source: Mol Biomed. 2024 Nov 1;5:48. doi: 10.1186/s43556-024-00212-z (PMC11527862; doi:10.1186/s43556-024-00212-z)

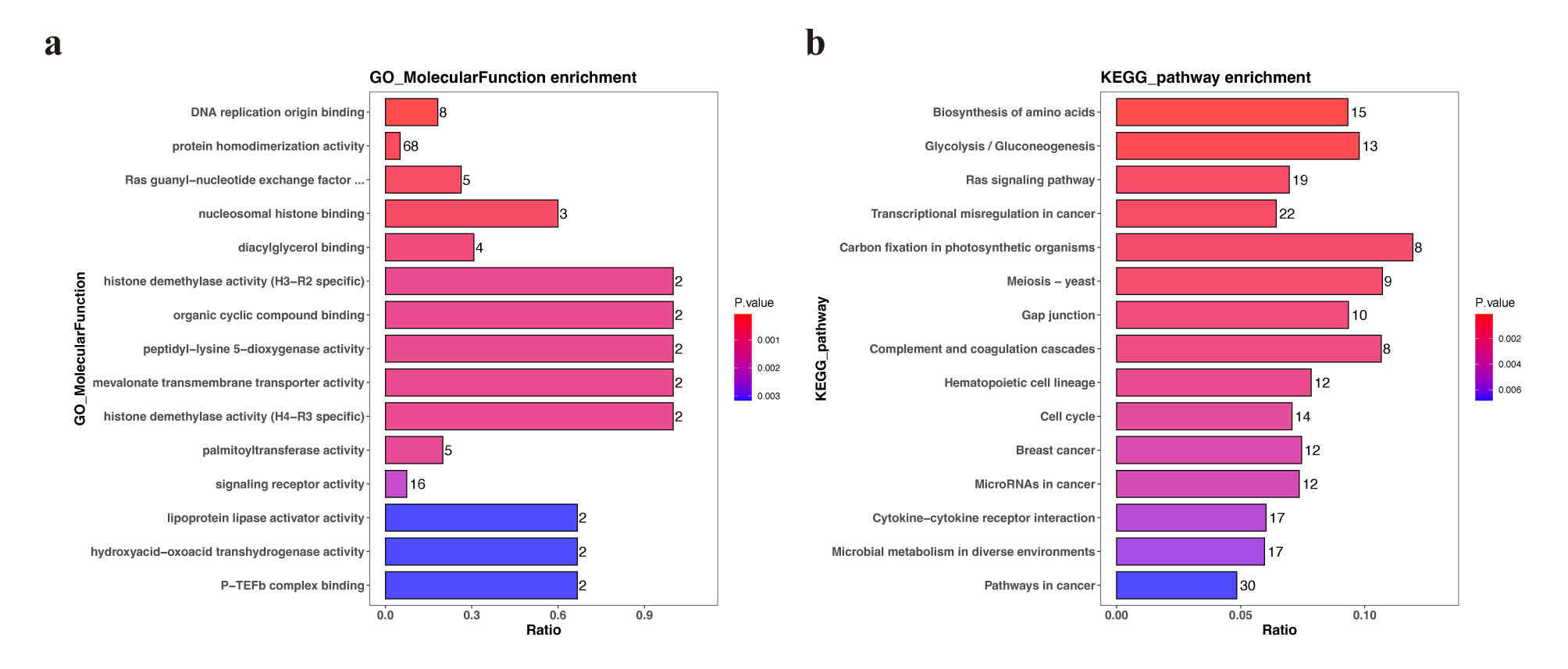

Supplement: Supplementary file 1 — Supplementary Material 1. [file 43556_2024_212_MOESM1_ESM.tif]

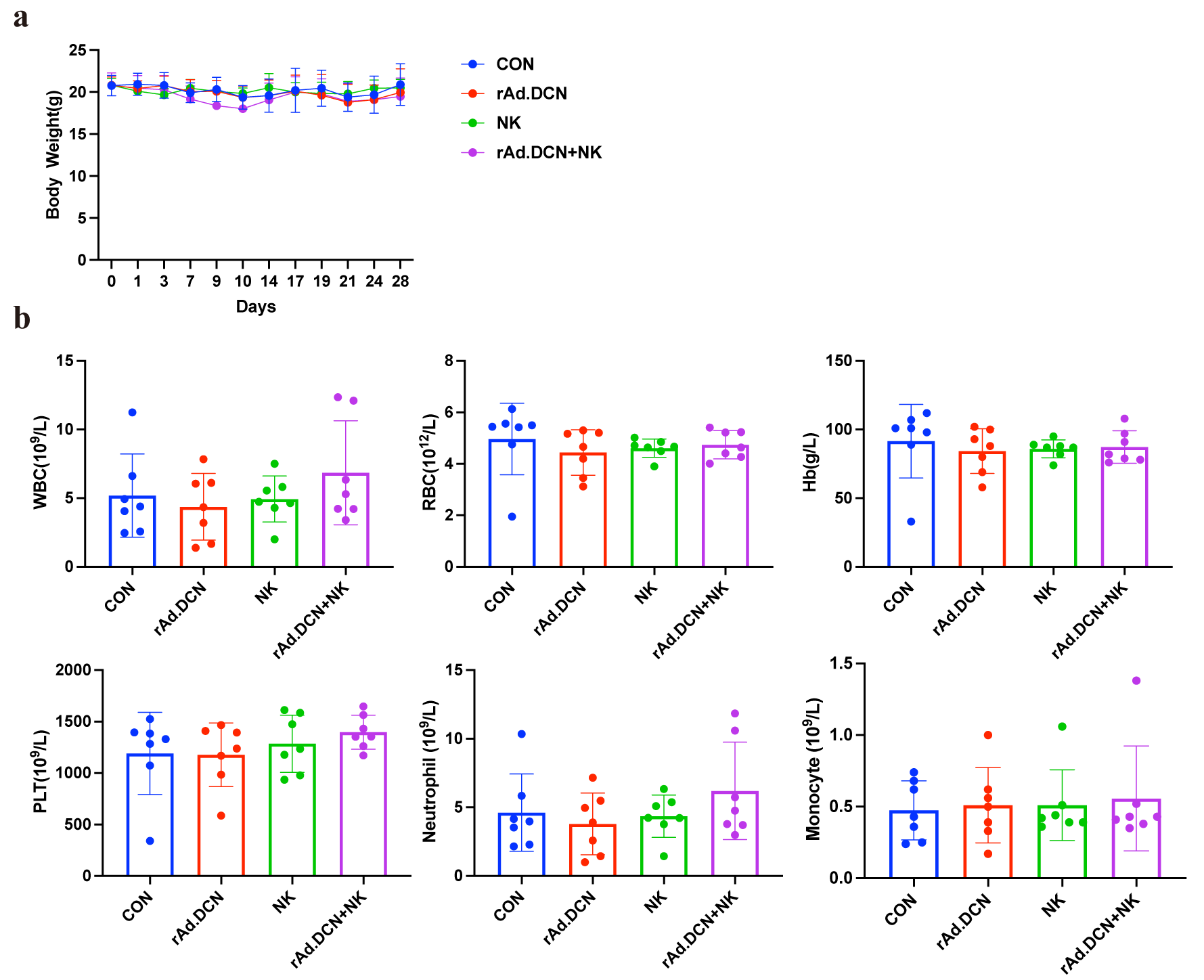

Supplement: Supplementary file 2 — Supplementary Material 2. [file 43556_2024_212_MOESM2_ESM.tif]
